# Impact on Patient Safety and Satisfaction of Implementation of an Outpatient Clinic in Interventional Radiology (IPSIPOLI-Study): A Quasi-Experimental Prospective Study

**DOI:** 10.1007/s00270-015-1069-4

**Published:** 2015-03-14

**Authors:** Jacob Lutjeboer, Mark Christiaan Burgmans, Kaman Chung, Arian Robert van Erkel

**Affiliations:** Department of Radiology, Leiden University Medical Center, Albinusdreef 2, P.O. Box 9600, 2300 RC Leiden, The Netherlands

**Keywords:** Interventional radiology, Outpatient care, Health care quality, Patient safety, Patient satisfaction

## Abstract

**Purpose:**

Interventional radiology (IR) procedures are associated with high rates of preparation and planning errors. In many centers, pre-procedural consultation and screening of patients is performed by referring physicians. Interventional radiologists have better knowledge about procedure details and risks, but often only get acquainted with the patient in the procedure room. We hypothesized that patient safety (PS) and patient satisfaction (PSAT) in elective IR procedures would improve by implementation of a pre-procedural visit to an outpatient IR clinic.

**Material and Methods:**

IRB approval was obtained and informed consent was waived. PS and PSAT were measured in patients undergoing elective IR procedures before (control group; *n* = 110) and after (experimental group; *n* = 110) implementation of an outpatient IR clinic. PS was measured as the number of process deviations. PSAT was assessed using a questionnaire measuring Likert scores of three dimensions: interpersonal care aspects, information/communication, and patient participation. Differences in PS and PSAT between the two groups were compared using an independent *t* test.

**Results:**

The average number of process deviations per patient was 0.39 in the control group compared to 0.06 in the experimental group (*p* < 0.001). In 9.1 % patients in the control group, no legal informed consent was obtained compared to 0 % in the experimental group. The mean overall Likert score was significantly higher in the experimental group compared to the control group: 2.68 (SD 0.314) versus 2.48 (SD 0.381) (*p* < 0.001).

**Conclusion:**

PS and PSAT improve significantly if patients receive consultation and screening in an IR outpatient clinic prior to elective IR procedures.

## Introduction

In 1964, Charles Dotter performed the first percutaneous transluminal angioplasty (PTA) in a patient with a superficial femoral artery stenosis [1]. This was the beginning of a new medical specialty: interventional radiology (IR). For years, transarterial therapies such as PTA and stent placement have been the hallmark of IR. Over the past two decades, many new IR procedures have been introduced for indications other than atherosclerotic occlusive disease. Thanks to technological innovations, the realm of IR now offers a wide variety of minimally invasive treatments such as uterine artery embolization, biliary stenting, percutaneous ablation, transarterial (chemo) embolization, radioembolization, vertebroplasty, and etcetera. In contrast to the technological revolution of IR, organization of patient care in many IR departments has seen limited change since the days of Charles Dotter. Many IR centers have not taken full responsibility for the care of patients and still rely on the referring physician to organize aspects of care other than the procedure itself. Such practise is questionable in a time where procedure complexity and indications have expanded to such an extent that few physicians other than the interventional radiologist will have sufficient insight into the potential benefits and harms of a procedure.

Studies have shown that IR procedures are associated with high rates of preventable errors related to pre-planning and patient preparation [2, 3]. Such errors may result in treatment delay or last-minute postponement and could jeopardize patient safety [2, 3]. Also, the way informed consent is currently obtained for many IR procedures raises legal concerns. In many centers, patients will only get acquainted with the interventional radiologist performing the procedure once they have arrived at the procedure room [4].

Improvements have been made in many hospitals by the introduction of IR safety checklists, as it has in our institution [2, 5]. Yet, we hypothesized that further improvements could be made if patients undergoing elective IR procedures would be screened and consented preoperatively in an IR outpatient clinic. We therefore conducted a prospective study with the aim to compare patient safety and patient satisfaction between patients who were subjected to a pre-procedural visit to an IR outpatient clinic (experimental group) and those who were not (control group).

## Methods

### Design

The study was conducted in accordance with the ethical standards of the institutional review board (IRB) and with the 1964 Helsinki declaration and its later amendments. Informed consent was waived by the IRB.

The study was designed as a single center, non-randomized, and prospective study. Patient safety and patient satisfaction were assessed prospectively in patients undergoing elective IR procedures. Outcomes were assessed in a group of patients before implementation of the IR outpatient clinic (control group) and then compared to those in a group of patients who were treated after implementation of the IR outpatient clinic and had made a visit to the clinic. The primary purpose of the study was to compare patient safety associated with elective IR procedures between the experimental group and the control group. The secondary purpose was to compare patient satisfaction between the two groups.

Power-analysis (Medclac version 12.4.0.0; Medcalc software) was based on a type 1 error of 0.05, a power of 80 % and the assumption that implementation of an IR outpatient clinic would lead to a 14 % reduction in the number of process deviations. This resulted in a calculated sample size of 220 patients with 110 patients in each group.

### Participants

**Fig. 1:**
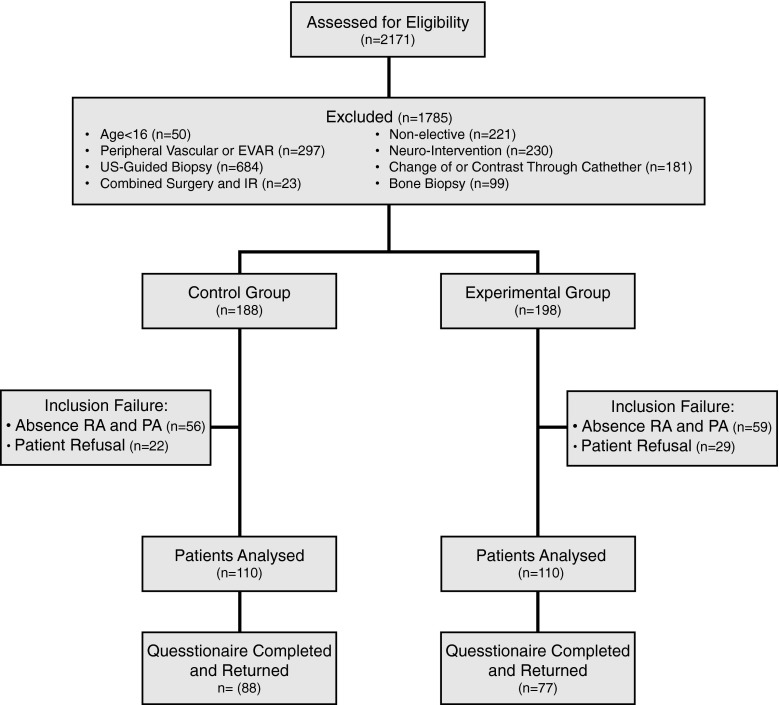
Flow diagram showing total number of patients screened, exclusion numbers and reasons, and per group analysis. *RA* research assistant, *PA* physician assistant

Patients undergoing an elective IR procedure during the study period were eligible if they were older than 16 years and mentally capable to fill out the Dutch questionnaire. Patients undergoing one of the following procedures were excluded: peripheral vascular intervention or endovascular aortic repair (EVAR), cerebral interventions, non-elective interventions, change of drainage catheter or contrast injection through a drainage catheter, combined surgical and IR procedure, ultrasound-guided biopsy, or bone biopsy (see Fig. 1). At the time of commencement of the study a close collaboration existed in our institution between interventional radiologists and vascular surgeons. Interventional radiologists were already involved in screening and consenting of patients in the vascular clinic, vascular surgeons were participating in peripheral vascular interventions in the angiography room and all EVARs were performed by a team of interventional radiologists and vascular surgeons. We therefore excluded patients undergoing peripheral vascular interventions or EVAR. The second category of patients was not included as cerebral interventions in our institution were already routinely preceded by outpatient consultation by a neuro-interventionalist. Ultrasound-guided biopsy and bone biopsy were excluded in order not to cause any diagnostic delay. Our institution is committed to a national program that guarantees a diagnosis within 48 h for 80 % of patients suspected to have one of 23 pre-defined cancer types.

Prior to their appointment for an elective IR treatment, patients were informed of the details and intent of the study by letter. Patients in the experimental group were invited for a visit to the outpatient clinic. All patients were asked to fill out a questionnaire after the procedure at a voluntary basis. If patients indicated that they were unwilling to visit the IR outpatient clinic or fill out the questionnaire, they were excluded from the study (Table 1).

**Table 1:** Baseline characteristics for control and experimental group

Characteristic	Control group (*n* = 110)	Experimental group (*n* = 110)	*p* value
Age (mean ± SD)	56.6 ± 16.1	57.9 ± 14.7	0.514
Sex (*N*)			0.050
Male	63	77	
Female	47	33	
Questionnaires response (*N* %)	88 (80.0 %)	77 (70.0 %)	
Type of procedure (*N* %)			0.011
Ablations	27 (24.5 %)	29 (26.5 %)	
Biopsy	33 (30.0 %)	43 (39.1 %)	
Drainages	15 (13.5 %)	4 (3.6 %)	
Embolization	15 (13.5 %)	24 (21.8 %)	
Central venous access	15 (13.5 %)	5 (4.5 %)	
Stents/PTA non arterial	5 (4.5 %)	5 (4.5 %)	

### Intervention

Patients in the experimental group were scheduled for an appointment in the IR outpatient clinic 2–14 days prior to the IR-procedure. During the appointment patients would be screened for risk factors and provided with information about the procedure by an interventional radiologist or physician assistant. In the same setting informed consent would be obtained. A key point list was used to ensure screening and consenting was performed adequately (see Table 2). All relevant matters discussed were recorded in the electronic patient records (EZIS, Chipsoft, The Netherlands).

**Table 2:** Key point information and outpatient screening list

Information	Discussed		
Procedure			
Indication	□ Yes	□ No	
Method of anaesthesia	□ Yes	□ No	
Procedure details explained	□ Yes	□ No	
Procedure length discussed	□ Yes	□ No	
Expected treatment outcome explained	□ Yes	□ No	
Complications			
Bleeding	□ Yes	□ No	□ N.A.
Infection	□ Yes	□ No	□ N.A.
Thrombus/embolus	□ Yes	□ No	□ N.A.
Neurogenic complications	□ Yes	□ No	□ N.A.
Non-Target	□ Yes	□ No	□ N.A.
Allergy	□ Yes	□ No	□ N.A.
Pneumothorax	□ Yes	□ No	□ N.A.
Other	□ Yes	□ No	□ If yes, specify:
Post-procedure			
Puncture site care	□ Yes	□ No	□ N.A.
Drain management	□ Yes	□ No	□ N.A.
Suture management	□ Yes	□ No	□ N.A.
Pain management	□ Yes	□ No	□ N.A.
Admission time	□ Yes	□ No	□ N.A.
Other	□ Yes	□ No	□ If yes, specify:
Screening	Checked		
Contra-indications	□ Yes	□ No	
Contrast allergy	□ Yes	□ No	
Renal function	□ Yes	□ No	
Anti-coagulation	□ Yes	□ No	
Other medication	□ Yes	□ No	
Other allergy	□ Yes	□ No	

Patients in the control group were not routinely screened or consented by medical IR staff prior to the procedure. Upon scheduling of an IR procedure, one of two IR administrative assistants would perform a pre-procedural check to verify whether blood tests showed any coagulopathy or renal insufficiency and whether anesthesiological support and specific tools were ordered as requested by the interventional radiologist. If the administrative assistant felt that blood tests were abnormal or missing, they would inform the interventional radiologist who would then contact the referring physician. The administrative assistant would also check whether the patient was using anti-coagulants and contact the patient by telephone to verify that instructions were given to temporarily stop the medication if deemed necessary. Prior to the procedure both the IR technician and interventional radiologist would assess different items of an IR safety checklist to ensure that the procedure could commence safely. Upon arrival at the procedure room, patients were asked whether the procedure and complication risks had been explained to them sufficiently. If a patient or the interventional radiologist felt that insufficient information had been provided, additional information was given. If the referring physician had recorded in the patient records that the diagnosis and prognosis of a disease had been discussed with the patient, the nature, aim, and risks of the procedure had been explained, alternative treatments had been discussed and informed consent had been obtained, the informed consent was considered to be sufficient (written informed consent is not mandated in the Netherlands).

### Outcome Assessment

Baseline characteristics that were recorded included age, sex and the type of procedure.

**Fig. 2:**
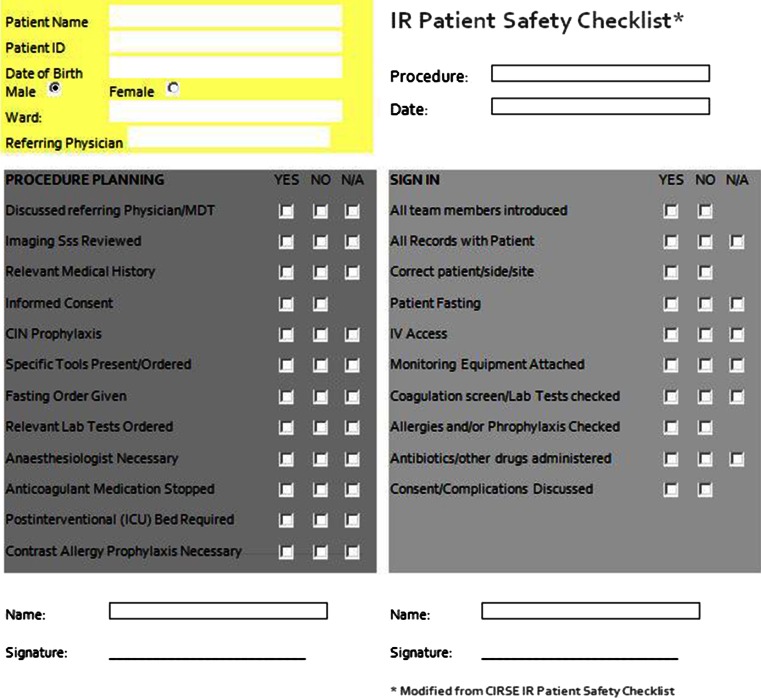
IR patient safety checklist

The primary outcome patient safety was assessed by measurement of the number of process deviations. A process deviation was defined as ‘an aspect of healthcare not executed correctly or not in accordance with IR protocols’. Process deviations were assessed using an IR safety checklist containing sections related to ‘pre-procedural planning’ and ‘sign-in’ (see Fig. 2). The checklist was derived from the IR patient safety checklist of the Cardiovascular and Interventional Radiological Society of Europe (CIRSE) [5]. Each section of the checklist was assessed by an independent research assistant or physician assistant at the time of the IR procedure. When there was some overlap between process deviations in two sections, only one process deviation could be scored in one of both sections. For example, a patient not having fastened before the procedure may have been a result of either a lack of information (Fasting Order Given in ‘Pre-procedural planning’) or the wrong instructions being given (Patient Fasting in ‘Sign-in’).

The secondary endpoint patient satisfaction was assessed by means of a validated questionnaire. The design and content of the questionnaire was based on the consumer quality index (CQI)-measurement instruments on outpatient care [6]. The questionnaire included 19 questions in Dutch measuring three dimensions: interpersonal aspects of care (5 items; *α* = 0.82; 1 factor), information and communication (7 items; *α* = 0.85; 1 factor) and patient participation patient (3 items; *α* = 0.63; 1 factor). Examples of the questionnaire were: “Did the doctor listen carefully to you?” and “Did the doctor explain things in an understandable way?”. The items were assessed on a 4-point Likert scale (strongly disagree, somewhat disagree, somewhat agree, and strongly agree). Four questions did not correlate with these three dimensions and were measured separately (see Table 5). The answers of individual patients were anonymized for interventional radiologists and referring physicians.

### Statistical Analysis

Data from the patient safety checklists and the questionnaires were analyzed using IBM SPSS Statistics 20 software (Chicago, IL, USA). Differences in baseline characteristics between the two groups were compared using a *χ* ^2^ test. For patient safety, differences in the mean process deviations between the two groups were tested using an independent *t* test.

Patient satisfaction scores were calculated for each dimension. The total Likert score for the three dimensions were calculated by adding up the score of each dimension (*α* > 0.60). The scores of the separate questions were assessed per question. Differences in the mean scores between the two groups were tested using an independent *t* test. All statistical analyses were two-tailed and values of *p* < 0.05 were considered significant.

## Results

### Participants

The study was conducted from April 2013 to January 2014. After inclusion of patients in the control group, a 4-week period was used to implement the IR outpatient clinic. Inclusion of patients in the experimental group commenced after these 4 weeks. The patient characteristics are listed in Table 1.

The number of female patients in the experimental group was significantly lower than in the control group: 33 versus 47 (*p* = 0.050). Also, there was a significant difference in the type of procedure between the groups (*p* = 0.011).

### Outcomes

#### Patient Safety

The differences in patient safety between the two groups are listed in Tables 3 and 4. The number of process deviations per patient was significantly lower in the experimental group compared to the control group: 0.06 versus 0.39 (*p* < 0.001). Significant differences in the number of process deviations were seen between the two groups in both sections of the IR safety checklist, ‘pre-procedural planning’ and ‘sign-in’. No process deviations were seen in ‘pre-procedural planning’ in the experimental group, whereas 0.22 process deviations per patient occurred in this section in the control group (*p* < 0.001). All patients in the experimental group had given legal informed consent, whereas 9.1 % (*n* = 10) of patients in the control group had not been consented adequately. Significant differences between the two groups were also seen in the section ‘sign in’: 0.06 process deviation per patient in the experimental group versus 0.17 in the control group (*p* = 0.021). Most process deviations in the section ‘sign in’ were related to the administration of antibiotics. Four patients in the experimental group and seven patients in the control group received prophylactic antibiotics prior to ablation of a liver tumor, while this was deemed unnecessary according to IR protocols. The doctors prescribing the antibiotics had followed the preoperative protocol used for surgical liver resection. Five patients in the control group arrived at the angiography room for a percutaneous gastrostomy without administration of prophylactic antibiotics as mandated by IR protocols.

**Table 3:** Process deviations per item of both sections for control and experimental groups

Characteristic	Control group (*n* = 110)	Experimental group (*n* = 110)
Items process deviations (*N*)		
Pre-procedural planning		
Discussed referring physician/MDT	2	0
Imaging studies reviewed	1	0
Relevant medical history	2	0
Informed consent/complications discussed	10	0
CIN prophylaxis	0	0
Specific tools present/ordered	4	0
Fasting order given	3	0
Relevant lab test ordered	0	0
Anaesthesiologist necessary	0	0
Anticoagulation medication stopped	2	0
Post interventional (ICU) bed required	0	0
Treatment limitation checked	0	0
Total pre-procedural planning	24	0
Sign in		
All records with patient	0	0
Correct patient/side/site	0	0
Patient fasting	3	0
IV access	3	0
Coagulation checked	1	1
CIN checked	0	2
Other lab tests checked	0	0
Allergies and/or prophylaxis checked	0	0
Antibiotics/other drugs administered	12	4
Total sign in	19	7
Total pre-procedural planning and sign in	43	7

**Table 4:** Overall number of process deviations per patient

Characteristic	Control group (*n* = 110)	Experimental group (*n* = 110)	*p* value
Process deviations (mean ± SD)			
Pre-procedural planning	0.22 ± 0.531	0.00 ± 0.000	<0.001
Sign in	0.17 ± 0.425	0.06 ± 0.245	0.021
Pre-procedural + sign in	0.39 ± 0.779	0.06 ± 0.245	<0.001

In the experimental group, there were no delays in treatment and 3 (2.7 %) postponements. In 2 of the 3 procedures that were postponed, the coagulation profile was unknown and blood tests had to be ordered before the procedure could be safely commenced. In the third patient, the creatinine or estimated glomerular filtration rate had not been determined prior to the procedure. In the control group, 19 (17.3 %) of the procedures where delayed to allow time to correct for process deviations. In 17 (15.5 %) procedures, the process deviation could not be corrected with the patient in the procedure room and the procedure was postponed to a later time or date. The causes for the postponement were: indication insufficiently discussed with the referring physician or in a multidisciplinary team (*n* = 2), missing relevant medical history (*n* = 2), absence of specific tools or material (*n* = 4), failure to stop anticoagulation medication (*n* = 2), fasting order not given (*n* = 3) or not correctly executed (*n* = 3), unknown coagulation profile (*n* = 1).

### Patient Satisfaction

The results of the questionnaires are summarized in Table 5. Total patient satisfaction showed a significance difference between the two groups in favour of the experimental group (*p* < 0.001). Significant improvement in patient satisfaction was seen after implementation of the IR outpatient clinic in all dimensions. The largest difference between the two groups occurred in the dimension ‘Information and communication’: an increase in the Likert scale score of 0.26 was seen after implementation of the IR outpatient clinic (*p* < 0.001).

**Table 5:** Questionnaire outcomes: average Likert score per dimensions of patient satisfaction, for separate questions and overall score per group

Characteristic	Control group (*n* = 88)	Experimental group (*n* = 77)	*p* value
Dimensions of patient satisfaction			
Interpersonal aspects	2.73 ± 0.402	2.89 ± 0.291	0.005
Information and communication	2.57 ± 0.571	2.83 ± 0.262	<0.001
Participation	2.38 ± 0.754	2.59 ± 0.613	0.067
Separate questions (mean ± SD)			
Interpersonal aspect			
Was doctor knowledgeable?	2.88 ± 0.357	2.87 ± 0.380	0.770
Information and communication			
Information was consistent with the actual treatment?	2.57 ± 0.770	2.75 ± 0.520	0.075
Information about duration of the treatment in accordance with the actual treatment?	2.34 ± 0.887	2.53 ± 0.644	0.120
Properly informed about preparation of the treatment	2.51 ± 0.919	2.65 ± 0.762	0.262
Overall patient satisfaction			
Patient satisfaction without separate questions	2.45 ± 0.398	2.67 ± 0.301	<0.001
Patient satisfaction	2.48 ± 0.381	2.68 ± 0.314	<0.001

## Discussion

In our study, we investigated the impact of implementation of a pre-procedural visit to an IR outpatient clinic for patients undergoing an elective IR procedure. The results show that patient safety and patient satisfaction improve significantly when patients receive preoperative screening and consultation in such a clinic.

In patients who were not seen in the clinic, a high rate of process deviations occurred: 0.39 per patient. After implementation of the clinic the number of process deviations was reduced to 0.06 per patient.

A study by Koetsier et al. has shown that the number of process deviations associated with IR procedures decreases when an IR safety checklist is used [2]. Such a checklist was used for all patients in our study. The use of the checklist allowed detection and correction of process deviations prior to commencement of the procedure in most patients in our study. Yet, it did not prevent delay and postponement of procedures in 17.3 and 15.5 % of patients, respectively. After implementation of an IR outpatient clinic, the percentages of delays and postponements were reduced to 0 and 2.7 %, respectively. The results of this study thus indicate that an IR outpatient clinic has additional value to IR safety checklists and implementation of such a clinic may lead to further improvements in patient safety.

Furthermore, implementation of the clinic resolved another important matter. Adequate informed consent had not been obtained prior to arrival of the patient at the procedure room in 9.1 % (*n* = 10) of patients in the control group. This high rate of inadequate informed consent in patients undergoing IR procedures is consistent with other reports. A survey by O’Dwyer et al. revealed that in 56 % of patients consent or re-consent for IR procedures is obtained in the procedure room and only 22 % of patients are consented in an outpatient clinic [4]. Requirements for legal informed consent vary per country, but the following three concepts of legal medical informed consent are widely accepted [7]. Firstly, medical treatment can only be started after a patient’s permission. Secondly, in order for the patient to make a decision, information about the patient’s medical condition, the treatment purposed and alternatives should be given in lay terms. Finally, the expected benefits and potential harms of the treatment should be explained to the patient. Legislation is usually not very specific on how these matters should be achieved, but obviously consent should be given in a proper manner, in an appropriate environment and in the presence of appropriate and relevant information [7]. Most people would affirm that consent for elective procedure should be obtained some time before the procedure and in an outpatient setting. Patients should be given time to think about the information provided to them and to read additional information from booklets or any other accessible medium. It seems reasonable to assume that interventional radiologists have better knowledge about details of an IR procedure than referring physicians and should therefore be the ones discussing relevant details with a patient. In our study, the number of patients without timely and adequate informed consent decreased to zero percent after implementation of an IR outpatient clinic.

Patient satisfaction is of paramount importance in building a good relationship between doctors and patients. In our study, patient satisfaction improved significantly by the implementation of an IR outpatient clinic. All aspects of patient care that were investigated (interpersonal aspects of care, information and communication and participation) improved after the IR clinic was implemented. The largest improvement in patient satisfaction was perceived in matters related to ‘patient information and communication’. The provided information on pre-procedural preparation, procedural details and the length of the procedure was also perceived to be more accurate in patients in the experimental group compared to the control group. This may not only have a positive effect on the relationship between doctors and patients, but may also have consequences for the legitimacy of the informed consent.

Over the last decades, IR has ridden the tidal wave of technological innovation to become a well-recognized medical specialty offering treatment for a variety of indications. Long gone are the days when interventional radiologists were the plumbers of the human vascular system with vascular surgeons being their main contractors. IR now caters to many different medical specialists offering a variety of therapies for many different indications. Despite the evolution of IR, in many centers the interventional radiologist has retained the traditional role between the stage scenes as a technician applauded for his catheter skills. A growing number of radiologists are now urging interventional radiologists to enter the stage as clinicians [8–10]. Our study shows that indeed both patient safety and patient satisfaction improve when IR takes on the responsibility to perform screening and provide information for patients undergoing IR procedures. It was Charles Dotter who said that the radiologist ‘who enters into treatment …can now play a key role, if he is prepared and willing to serve as a true clinician’ [1]. It is time for interventional radiologists to pay tribute to the father of IR by following his advice. This will also require diagnostic colleagues and hospital administrators to recognize the role of interventional radiologists as clinicians, allocating them time to perform the duties that come with it.

Our study has some limitations. Firstly, we were not able to account for the Hawthorne effect [11]. IR staff may have enhanced their efforts to reduce process deviations or to satisfy patients, knowing that they were being observed. Secondly, regression-to-the-mean may have had impact on the study results. Thirdly, the impact of the IR outpatient clinic was assessed in a quasi-experimental experiment. Thus, it is possible that the observed changes were to some extend affected by changes in time. Yet, the study period was only 7 months during which only minimal changes in policy and IR staff occurred. Fourthly, we measured a surrogate outcome, process deviations, to assess patient safety. A study comparing the complication rate between the experimental and control group would have required a much larger sample size. The majority of process deviations in the control group could be corrected before commencement of the procedure, but not without causing delay or postponement in many patients. Finally, we excluded patients undergoing peripheral vascular interventions, EVAR or neuro-interventions from our study for reasons explained above. These patients make up a large portion of all IR patients. Although our study results cannot automatically be extrapolated to these patients, it seems reasonable to assume that similar principles apply in these subgroups of patients. We acknowledge the fact that practises may vary from country to country and even from institution to institution. It may therefore not be possible to extrapolate our study findings to all institutions, but we believe the outcomes of our study to be applicable to many IR centers.

In conclusion, our study shows that the number of process deviations associated with elective IR procedures can be significantly reduced when patients are consulted in an IR outpatient clinic prior to the procedure. Also, by providing pre-procedural patient consultation in an outpatient setting IR can improve the satisfaction of patients. More patients will perceive the pre-procedural information provided by them as adequate and the number of patients in whom informed consent is inadequate can be reduced to zero. After the completion of our study, we have implemented a visit to the IR outpatient clinic for patients undergoing elective radiological interventions of moderate to high complexity. Patients undergoing elective procedures of low complexity, such as routine biopsies, venous catheters or drainages, are receiving telephone consultation.

## References

[1] Dotter CT (1980). Transluminal angioplasty: a long view. Radiology.

[2] Koetser IC, de Vries EN, van Delden OM, Smorenburg SM, Boermeester MA, van Lienden KP (2013). A checklist to improve patient safety in interventional radiology. Cardiovasc Intervent Radiol.

[3] Morbi AH, Hamady MS, Riga CV, Kashef E, Pearch BJ, Vincent C (2012). Reducing error and improving efficiency during vascular interventional radiology: implementation of a preprocedural team rehearsal. Radiology.

[4] O’Dwyer HM, Lyon SM, Fotheringham T, Lee MJ (2003). Informed consent for interventional radiology procedures: a survey detailing current European practice. Cardiovasc Intervent Radiol.

[5] Lee MJ, Fanelli F, Haage P, Hausegger K, Van Lienden KP (2012). Patient safety in interventional radiology: a CIRSE IR checklist. Cardiovasc Intervent Radiol.

[6] Delnoij DM, Rademakers JJ, Groenewegen PP (2010). The Dutch Consumer Quality Index: an example of stakeholder involvement in indicator development. BMC Health Serv Res.

[7] Cannavale A, Santoni M, Mancarella P, Passariello R, Arbarello P (2013). Malpractice in radiology: what should you worry about?. Radiol Res Pract.

[8] Murphy TP (2005). Introduction to clinical interventional radiology. Semin Intervent Radiol.

[9] Soares GM, Murphy TP (2005). Clinical interventional radiology: parallels with the evolution of general surgery. Semin Intervent Radiol.

[10] American College of Radiology; American Society of Interventional and Therapeutic Neuroradiology; Society of Interventional Radiology (2006). Practice guideline for interventional clinical practice. J Vasc Interv Radiol.

[11] Fernald DH (2012). An assessment of the Hawthorne Effect in practice-based research. J Am Board Fam Med.

